# BRAIN CLEARANCE SYSTEM ALTERATION AND COGNITIVE DECLINE IN AFRICAN AMERICANS WITH POOR SLEEP QUALITY

**DOI:** 10.1093/geroni/igae098.1726

**Published:** 2024-12-31

**Authors:** Karen Lincoln, Bryan Gaines, Kaushik Golithadka

**Affiliations:** University of California Irvine, Irvine, California, United States; University of California Irvine, Irvine, California, United States; University of Southern California, Los Angeles, California, United States

## Abstract

African Americans have 2-3 times the prevalence of Alzheimer’s disease (AD) than Whites, making this a significant health disparity in the US. Healthy sleep plays a critical role in providing protection from cognitive decline and AD. Unfortunately, African Americans have significantly poorer indicators of sleep quality than Whites. Moreover, the mechanism whereby sleep drives AD and related cognitive decline is not well understood, in part because causal links are yet to be established by human studies. One possible mechanism linking sleep to AD is the glymphatic system, a network of perivascular spaces (PVS) that support waste clearance. This study used ultra-high field magnetic resonance imaging technology, novel PVS mapping technology, wrist actigraphy and sleep assessments, cognitive assessments and regression analysis to investigate how alteration of the glia-lymphatic system associated with sleep contributes to cognitive decline in 61 community-dwelling African Americans (age 45+). African American who slept more and spent more time in bed had a higher risk for cognitive impairment as indicated by lower MMSE scores. We also found that African Americans with larger PVS volume (more metabolic waste in the brain) had lower cognitive scores. The effect of sleep quality on cognitive status was mediated by PVS, such that African Americans who spent more time in bed had larger PVS (more waste and fluid) and lower cognitive scores. Findings from this study advance our understanding of the role of sleep and brain clearance in cognitive decline in African Americans, and identify PVS as a novel marker for cognitive decline.

